# Cr$_2$AlN and the search for the highest temperature superconductor in the M$_2$AX family

**DOI:** 10.1038/s41598-023-33517-0

**Published:** 2023-04-21

**Authors:** E. Karaca, P. J. P. Byrne, P. J. Hasnip, M. I. J. Probert

**Affiliations:** 1 Department of Physics, University of York, York, YO10 5DD UK; 2 Sakarya University, Biomedical, Magnetic and Semiconductor Materials Research Center (BIMAS-RC), 54187 Sakarya, Turkey

**Keywords:** Condensed-matter physics, Superconducting properties and materials, Materials science

## Abstract

We have developed a high-throughput computational method to predict the superconducting transition temperature in stable hexagonal M$_2$AX phases, and applied it to all the known possible choices for M (M: Sc, Ti, V, Cr, Mn, Fe, Y, Zr, Nb, Mo, Lu, Hf and Ta). We combine this with the best candidates for A (A: Al, Cu, Ge and Sn ) and X (X: C and N) from our previous work, and predict T$_c$ for 60 M$_2$AX-phase materials, 53 of which have never been studied before. From all of these, we identify Cr$_2$AlN as the best candidate for the highest T$_c$, and confirm its high T$_c$ with more detailed density functional theory electron-phonon coupling calculations. Our detailed calculations predict $T_c$ = 14.8 K for Cr$_2$AlN, which is significantly higher than any $T_c$ value known or predicted for any material in the M$_2$AX family to date.

## Introduction

The MAX phases are a fascinating family of materials, normally expressed as M$_{n+1}$AX$_n$, n = 1, 2, 3, etc., where M is an early transition metal, A is an element from groups 12–16, and X is usually B, C or N^1–3^. They combine the metal-like properties of high electrical and thermal conductivity, machinability and mechanical strength^4–11^ with the ceramic-like properties of good mechanical properties at high temperature and very good corrosion and oxidation resistance, high hardness, high creep lifetime^12–19^. These properties make them suitable for use as advanced materials in a variety of technologies and under extreme conditions. Jeitschko et al. reported on the manufacturing and characterization of Ti$_2$AlN^1^ in 1963, from which the hexagonal M$_2$AX phase family developed. There are about 60 different M$_2$AX phases that have been synthesised^20^, but only 10 of them have been experimentally proven to be superconductors: Mo$_2$GaC (with $T_c$ = 4.0 K)^21^, Nb$_2$SC (5.0 K)^22^, Nb$_2$AsC (2.0 K)^23^, Nb$_2$SnC (7.8 K)^24^, Ti$_2$InC (3.1 K)^25^, Nb$_2$InC (7.5 K)^26^, Ti$_2$InN (7.3 K)^27^, Ti$_2$GeC (9.5 K)^28^, Lu$_2$SnC (5.2 K)^29^ and Nb$_2$GeC (10.0 K)^30^. Nb$_2$GeC has the highest experimental $T_c$ = 10 K of any of these compounds currently known^30^. In a recent paper on Ti-based M$_2$AX phases, we predicted Ti$_2$AlN would have $T_c$ = 13.0 K, which was the highest superconductivity temperature of all the materials studied^3^.

The purpose of this study is to increase the maximum $T_c$ in superconducting M$_2$AX phases by considering the effect of varying M. In our previous work, we first considered the effect of changing A in the Nb-based carbides, and created a high-throughput screening approach based upon the Fröhlich model, using Eliashberg calculations and experimental data for T$_c$ of 4 phases which were already known to be superconducting. We examined 9 new choices for A and found A = Al yielded the highest $T_c$ = 6.7 K^2^ of these. In our next study of Ti-based materials, we used the same approach to study 42 phases, of which only 3 were already known to be superconducting, and predicted that A = Al and X = N would have the highest $T_c$ = 13.0 K^3^.

In this paper, we study the effect of changing M. This significantly increases the number of possible phases, and is much more disruptive to the electronic and phononic structure of the material. Experimentally, the list of elements that can substitute for M in stable M$_2$AX phases is currently known to be Sc, Ti, V, Cr, Mn, Fe, Y, Zr, Nb, Mo, Lu, Hf and Ta.

Of the 10 different M$_2$AX phases that are known to be superconducting, the effect of changing M for fixed A and X can be seen by considering Ti$_2$GeC vs Nb$_2$GeC, and for Nb$_2$SnC vs Lu$_2$SnC. Hence we first studied these four materials using detailed Eliashberg calculations and compared to known experimental $T_c$ values. We then extended this with detailed Eliashberg calculations to include additional phases M$_2$AC (M: Nb, Ti, Mo and Lu; A: Ge and Sn), 3 of which we have already presented in previous works^2,3^, and the 5 new sets of data are presented in the Supplementary Information. This was then used to generate a high-throughput screening model for M$_2$AC (A: Ge and Sn). We then used this model on the full set of 13 M elements (with both A: Ge and Sn) and found that M = Cr was predicted to have the highest $T_c$ both times. Combining the insights from our previous studies^2,3^ on the effect of changing A and X for fixed M, this suggested that Cr$_2$AlN should be the best combination. To be robust we used this initial screening to identify the 5 best values of M (M: Ti, Cr, Fe, Nb and Mo) for further study in case the ranking changed as we varied A and X.

Sun et al.^31^ investigated the structural and electronic properties of Cr$_2$AlN using ab initio calculations in 2005. The structural, electronic, and elastic properties of Cr$_2$AlN have been analyzed by using density functional theory with the generalized gradient approximation (GGA)^32–34^. However, no ab initio works for the phonon properties of Cr$_2$AlN have been discovered, even though many physical properties, such as electrical and thermal resistivity, thermal expansion, and superconductivity, are determined by phonons and their interactions with electrons. To estimate electron–phonon matrix elements for Cr$_2$AlN, we combined the linear response approach^35,36^ and the Migdal–Eliashberg approach^37,38^ to calculate the Eliashberg spectral function of Cr$_2$AlN using the phonon density of states and electron–phonon matrix elements. Integrating the Eliashberg spectral function produced the average electron–phonon coupling parameter and logarithmic average phonon frequency for Cr$_2$AlN. These physical characteristics are used to calculate the superconducting transition temperature using the Allen–Dynes modified McMillan formula^39–41^. In our previous studies^2,3^ on superconductivity in some M$_2$AX phases using the same techniques, we observed that the Migdal-Eliashberg predictions agreed with experimental $T_c$ values to within ± 1 K.

## Methods

The quantum mechanical calculations were performed using the Quantum–Espresso ab initio simulation package^35,36,42^ in which the plane-wave pseudopotential method is applied. The electron-ion interactions are modeled using ultrasoft pseudopotentials^43^. The Perdew–Burke–Ernzerhof (PBE) scheme is used to approximate the exchange-correlation functional^44^. The plane-wave basis cut-off for all calculations is set to 60 Ry ($\sim$ 812 eV) and the Brillouin zone integration used the Monkhorst–Pack^45^ scheme with ($36\times 36 \times 8$) **k**-mesh (maximum spacing of 0.01 $\times 2\pi ~{\mathring{\text {A}}}^{-1}$), whereas the electronic and Fermi surface calculations utilise a denser ($40\times 40 \times 10$) **k**-mesh. We also allowed for the emergence of magnetic properties in the Fe-based materials, but no evidence for spin polarisation was found.

The Brillouin zone integration for phonons used a ($4\times 4 \times 4$) **q**-mesh and twelve dynamical matrices by symmetry for phonon calculations. From this, the electron–phonon interaction can be calculated and hence $T_c$.

The electron–phonon interaction has been investigated with the linear response theory^35,36^ approach to the Migdal–Eliashberg theory^37,38^. The scattering of an electron in state ${|{\textbf{k}n}\rangle }$ to state ${|{\mathbf{k+q}m}\rangle }$ due to the phonon mode $\omega _{\textbf{q}j}$ perturbation is given by the electron-phonon matrix elements $\textrm{g}^{\textbf{q}j}_{(\textbf{k}+\textbf{q})m;\textbf{k}n}$ which have been estimated self-consistently by the linear response theory^35,36^.

The electron–phonon matrix element *g* is given as^46–50^

1 $$\begin{aligned} g^{{{\varvec{q}}}j}_{({{\varvec{k}}+ {\varvec{q}}})m;{{\varvec{k}}}n} = \sqrt{\frac{\hbar }{2\omega _{{{\varvec{q}}}j}}} {\langle { {{\varvec{k}} + {\varvec{q}}},m|\Delta V^{SCF}_{{\varvec{q}}, j}|{{\varvec{k}}},n}\rangle }, \end{aligned}$$

where ${|{{{\varvec{k}}}, n}\rangle }$ is the Bloch electron eigenstate with wavevector ${{\varvec{k}}}$ and band index *n*; $\Delta V^{SCF}_{{\varvec{q}} j}$^50^ is the derivative of the self-consistent effective potential with respect to the atomic displacements caused by a phonon of frequency $\omega _{{\varvec{q}},j}$ with wave vector ${{\varvec{q}}}$ and branch index *j*.

From these matrix elements, the phonon linewidth can be calculated by averaging the electron–phonon interaction over the Fermi surface:

2 $$\begin{aligned} \gamma _{\textbf{q}j}=2\pi \omega _{\textbf{q}j} \sum _{\textbf{k} nm}|\textrm{g}^{\textbf{q}j}_{(\textbf{k}+\textbf{q})m;\textbf{k}n}|^2 \delta (\varepsilon _{\textbf{k}n}-\varepsilon _{F})\delta (\varepsilon _{(\textbf{k}+\textbf{q})m}-\varepsilon _{F}). \end{aligned}$$

The Dirac delta functions restrict the initial and final states to the Fermi surface as the phonon energy is small in comparison.

The **q**-dependent electron-phonon coupling parameter is given by

3 $$\begin{aligned} \lambda _{\textbf{q}j}= \frac{\gamma _{\textbf{q}j}}{\pi \hbar N(\varepsilon _{F})\mathrm{\omega }^{{2}}_{{{\varvec{q}}}j}}. \end{aligned}$$

This can be used to measure the contribution of each phonon branch to the total electron–phonon coupling.

An alternative route to the average electron–phonon coupling parameter $\lambda$ is via the Eliashberg spectral function $\alpha ^2$F($\omega$). This can be defined in terms of the phonon linewidth $\gamma _{\textbf{q}j}$ as

4 $$\begin{aligned} \alpha ^2 F(\omega )=\frac{1}{2\pi N(E_{F})} \sum _{\textbf{q}j}\frac{\gamma _{\textbf{q}j}}{\hbar \omega _{\textbf{q}j}}\delta \left( \omega -\omega _{\textbf{q}j}\right) , \end{aligned}$$

and then the average electron–phonon coupling parameter $\lambda$ and the logarithmically averaged phonon frequency $\omega _{ln}$ can be derived from the integration of the Eliashberg spectral function:

5 $$\begin{aligned} \lambda= & {} 2\int \frac{\alpha ^{2}F(\omega )}{\omega }d\omega . \end{aligned}$$

6 $$\begin{aligned} \omega _{ln}= & {} \exp \left( 2\lambda ^{-1}\int _{0}^{\infty } \frac{d\omega }{\omega }\alpha ^2F(\omega )\ln \omega \right) . \end{aligned}$$

The Allen–Dynes modified McMillan equation can then be used to determine the superconducting critical temperature $T_c$^40,41^,

7 $$\begin{aligned} T_{c}=\frac{\omega _{\textrm{ln}}}{1.2}\textrm{exp} \left( -\frac{1.04(1+\lambda )}{\lambda -\mu ^{*}(1+0.62\lambda )}\right) , \end{aligned}$$

where $\mu ^{*}$ represents the effective screened Coulomb repulsion parameter. In most studies, the value of the value of $\mu ^{*}$ varies between 0.10 and 0.16^40,41^.

The calculation of $T_c$ is very computationally intensive, as the electron–phonon coupling requires a finely sampled Fermi surface, which leads to a large number of matrix elements that need to be calculated, making it unsuitable for high-throughput screening of potential superconductors. In a previous paper^2^ on Nb-based M$_2$AX phases we showed that changing A in M$_2$AC was comparable to the superconducting isotope effect, and that a simple model based on the Fröhlich^51^ theory of the isotope effect provided an effective basis for a high-throughput screening technique of these materials, with

8 $$\begin{aligned} T_c = \alpha \frac{N(E_{F})}{\sqrt{M}} - T_0, \end{aligned}$$

where M is the mass of a formula unit, N(E$_F$) is the electronic density of states at the Fermi energy E$_F$, and T$_0$ and $\alpha$ are linear fit parameters. For superconductivity to occur, the critical value for this model is $N(E_F)/\sqrt{M} > T_0/\alpha$. This functional form is an approximation to the simplified BCS equation^52^.

In a subsequent paper^3^ on Ti-based M$_2$AX phases we showed that the same approach was successful when both A and X were varied, and that 3 distinct trends were clear for X = C, N and B.

The advantage of Eq. 8 is that N(E$_F$) can be calculated in around 1 h using 40 cores of a modern supercomputer, whereas the electron–phonon matrix element calculation typically takes over 200 h with the same computing resource per material. When combined with the observed trend in $T_c$ vs. $N(E_F)/\sqrt{M}$, it can be used in a high-throughput search to predict the superconducting transition temperatures of candidate materials.

In this work, we consider the effect of changing M. This is expected to be much more disruptive to electron–phonon coupling, as our earlier studies have shown that N(E$_F$) is dominated by electronic contributions from M, and the most important part of the electron–phonon coupling parameter ($\lambda$) comes from low frequency phonons, which are also dominated by M.

## Results

### Superconducting $T_c$ results

**Figure 1:**
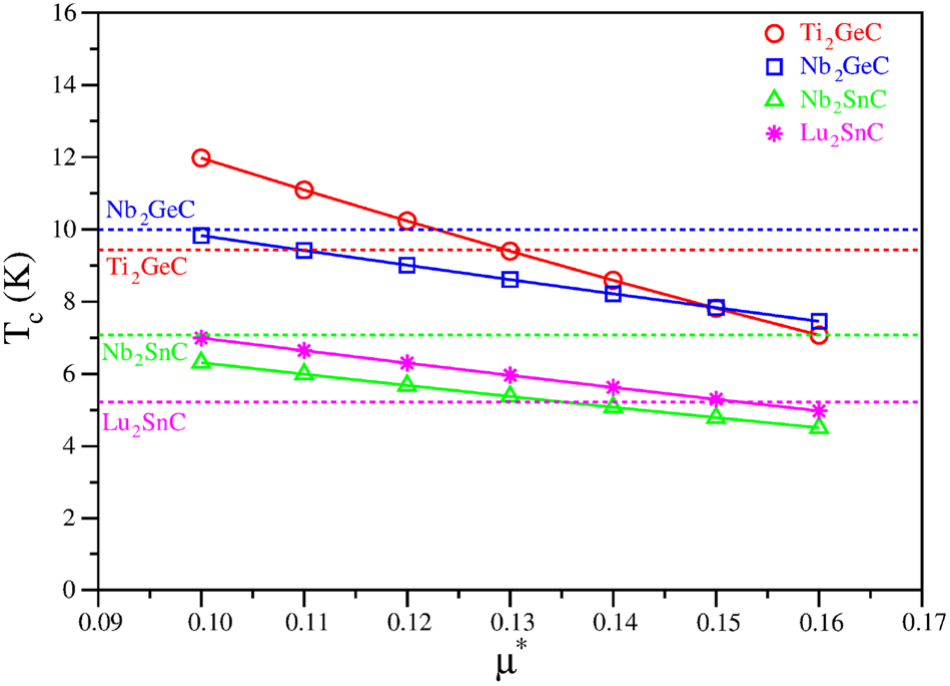
Calculated $T_c$ for seven different values of Coulomb repulsion constant ($\mu ^*$). The experimental values of Ti$_2$GeC, Nb$_2$GeC, Nb$_2$SnC and Lu$_2$SnC are shown as horizontal red, blue, green and magenta dashed lines, respectively.

The first part of this study was to establish the optimal value of $\mu ^{*}$ by calculating $T_c$ values using the Eliashberg theory for the 4 M$_2$AC phases (M: Ti and Nb; A = Ge) and (M = Nb and Lu; A = Sn) for which the experimental values of $T_c$ are known. We consider the usual range of $0.10<= \mu ^{*} <= 0.16$ and compare to known experimental data in Fig. 1. The best $\mu ^{*}$ value depends on M as expected, but overall, $\mu ^{*}$ = 0.13 is most reliable with a worst case error in $T_c$ of ± 1.5 K, and for most materials, the error is significantly better than that. We also note that fixing $\mu ^{*}$ may lead to small errors in the rank ordering of $T_c$ with varying M, but that the general trends of higher or lower $T_c$ are reliable. Fixing $\mu ^{*}$ = 0.13 is also consistent with our previous work^3^.

**Figure 2:**
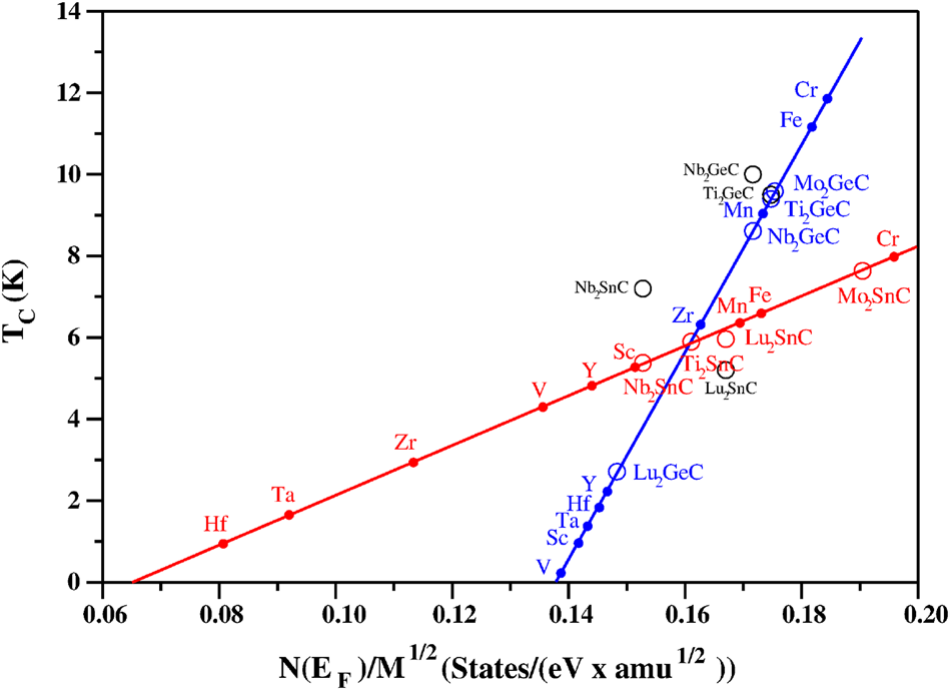
$T_c$ for M$_2$AC (M: Sc, Ti, V, Cr, Mn, Fe, Y, Zr, Nb, Mo, Lu, Hf and Ta; A: Ge and Sn). The results for $T_c$ calculated using $\mu ^*=$ 0.13 using Migdal-Eliashberg theory are presented as blue (A = Ge) and red (A = Sn) open circles, and the corresponding experimental data are displayed as black circles, with linear best fit to the theoretical values in blue and red lines. Red and blue filled circles represent a simple Fröhlich model for estimating superconducting transition temperature $T_c$. Full data in Tables 1 and 2.

The next step was to establish the high-throughput screening model, by expanding the range of phases (M: Ti, Nb, Mo and Lu; A: Ge and Sn) studied using the Eliashberg theory. This confirmed the linear relation between $T_c$ and N(E$_F$)/$\sqrt{M}$ and by fitting to Eq. 8, values for $\alpha$ and $T_0$ were determined. This showed that the Fröhlich model was valid in this work. The $T_c$ results are given in Table 1 and shown in Fig. 2, where blue and red squares show that A = Ge and A = Sn naturally separate into two separate classes, as shown by two straight lines. In addition, the available experimental $T_c$ values are shown in black circles. Detailed Eliashberg results for the phases not studied in our earlier works are presented in the Supplementary Information.

**Figure 3:**
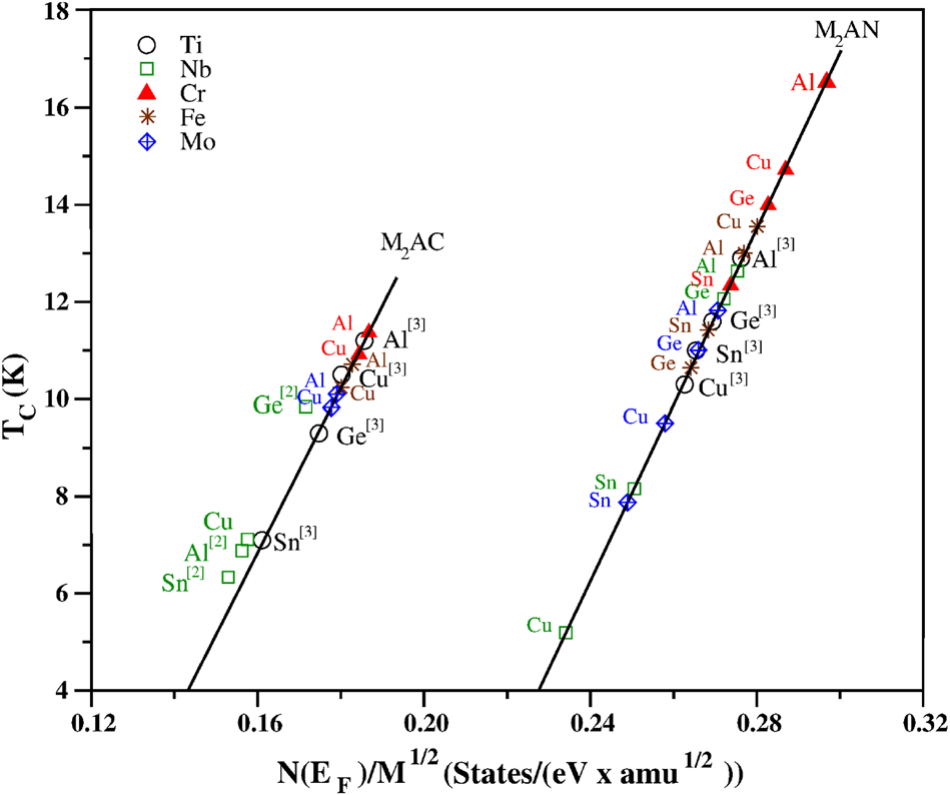
$T_c$ for M$_2$AX (M: Ti, Cr, Fe, Nb and Mo; A: Al, Cu, Ge and Sn; X: C and N). New data in this paper for M = Ti, Nb, Cr, Fe, and Mo are shown as black circles, green squares, red filled triangles, brown stars and blue diamonds, respectively. Data from our recent studies^2,3^ on Nb$_2$AC and Ti$_2$AX are shown in brackets. Full data in Table 3.

**Table 1:** Superconducting temperature values ($T_c$ in K) for M$_2$AC (M:Ti, Nb, Mo and Lu, A: Ge and Sn) in experiment, predicted using the Fröhlich model and calculated by Migdal–Eliashberg (ME) theory (with $\mu ^{*}=$ 0.13).

Phase	$T_{c}$ (exp)	$T_{c}$ ( Fröhlich)	$T_{c}$ (full ME theory)
Ti$_2$GeC	9.5^28^	9.3^3^	9.4^3^
Nb$_2$GeC	10.0^30^	8.5	8.6*
Mo$_2$GeC		9.6	9.6
Lu$_2$GeC		2.5	2.7
Ti$_2$SnC		7.1^3^	5.9
Nb$_2$SnC	7.2^53^	5.3	5.4*
Mo$_2$SnC		7.6	7.6
Lu$_2$SnC	5.2^29^	6.2	5.9

*This value was calculated with $\mu ^{*}=$ 0.13 whilst the corresponding value in ref.^2^ had $\mu ^{*}=$ 0.10.

Having established our high-throughput model, we can now predict $T_c$ in new M$_2$AX phases from just N(E$_F$) and the mass. The model predictions of $T_c$ for M$_2$AC (where M: Sc, Ti, V, Cr, Mn, Fe, Y, Zr, Nb, Mo, Lu, Hf, and Ta; A: Ge and Sn) are given in Table 2 and included in Fig. 2. Of these materials, 18 have no previously reported $T_c$ studies. Our predictions suggest that Cr-based phases have the highest $T_c$ for both families for M$_2$AC (A: Ge and Sn).

**Table 2:** Superconducting temperature values of all the different M$_2$AC (A: Ge and Sn) phases screened by the high-throughput Fröhlich model.

Phase	$N(E_F)$ (states/eV)	$\sqrt{M}$ (amu$^{0.5}$)	$T_{c}$ (K)
Sc$_2$GeC	2.65	18.7	0.9
V$_2$GeC	2.69	19.3	0.2
Cr$_2$GeC	3.58	19.4	11.7
Mn$_2$GeC	3.43	19.7	9.1
Fe$_2$GeC	3.61	19.8	11.2
Y$_2$GeC	3.35	22.9	2.1
Zr$_2$GeC	3.76	23.1	6.3
Hf$_2$GeC	4.31	29.7	1.8
Ta$_2$GeC	4.29	29.8	1.4
Sc$_2$SnC	3.22	21.0	5.3
V$_2$SnC	2.92	21.6	4.2
Cr$_2$SnC	4.25	21.7	7.9
Mn$_2$SnC	3.70	21.9	6.3
Fe$_2$SnC	3.81	22.0	6.5
Y$_2$SnC	3.59	24.8	4.8
Zr$_2$SnC	2.81	25.0	2.9
Hf$_2$SnC	2.49	31.2	0.8
Ta$_2$SnC	2.90	31.4	1.6

As a general trend, across the 4 families of M$_2$AX phases previously studied, (Nb$_2$AC, Ti$_2$AB, Ti$_2$AC and Ti$_2$AN) the best $T_c$ results were found with A = Al, Cu, Ge or Sn and X = C or N. In addition to M = Cr, there are 4 other choices (M: Ti, Fe, Nb and Mo) that have consistently high $T_c$ and should also be considered in case of a change in ranking when varying A and X, and effect of fixed $\mu ^{*}$. We therefore extended the range of phases considered and calculated N(E$_F$) for 40 phases to predict the $T_c$ of M$_2$AX (M: Ti, Cr, Fe, Nb and Mo; A = Al, Cu, Ge and Sn; X = C and N) of which 23 have not been reported before in this work or elsewhere. The results are given in Table 3 and shown in Fig. 3.

**Table 3:** Superconducting temperature values of M$_2$AC (M: Ti, Cr, Fe, Nb and Mo; A: Al, Cu, Ge and Sn; X: C and N) phases screened by the high-throughput Fröhlich model. Predicted $T_c$ for Nb$_2$AC (A: Al, Cu, Ge and Sn) and Ti$_2$AX (A: Al, Cu, Ge and Sn; X: C and N) are taken from our previous publications^2, 3^. The new predicted $T_c$ values based on the results in this paper are given in bold.

Phase	$N(E_F)$ (states/eV)	$\sqrt{M}$ (amu$^{0.5}$)	$N(E_F)$/$\sqrt{M}$ (states/(eV amu$^{0.5}$))	$T_{c}$ (K)
Ti$_2$AlC	3.05^3^	16.4^3^	0.1857	11.2^3^
Ti$_2$CuC	3.34^3^	18.5^3^	0.1802	10.5^3^
Ti$_2$GeC	3.32^3^	19.0^3^	0.1747	9.3^3^
Ti$_2$SnC	3.43^3^	21.3^3^	0.1610	7.1^3^
Cr$_2$AlC	3.13	16.9	0.1851	**11.1**
Cr$_2$CuC	3.48	18.9	0.1836	**10.7**
Fe$_2$AlC	3.17	17.4	0.1826	**10.5**
Fe$_2$CuC	3.51	19.4	0.1814	**10.4**
Nb$_2$AlC	3.31^2^	21.2^2^	0.1561	6.9^2^
Nb$_2$CuC	4.14	22.9	0.1575	**7.1**
Nb$_2$GeC	3.99^2^	23.3^2^	0.1716	9.8^2^
Nb$_2$SnC	3.84^2^	25.2^2^	0.1527	6.3^2^
Mo$_2$AlC	3.83	21.5	0.1782	**9.9**
Mo$_2$CuC	4.09	23.1	0.1768	**9.8**
Ti$_2$AlN	4.57^3^	16.5^3^	0.2762	12.9^3^
Ti$_2$CuN	4.89^3^	18.6^3^	0.2627	10.3^3^
Ti$_2$GeN	5.14^3^	19.1^3^	0.2693	11.6^3^
Ti$_2$SnN	5.67^3^	21.4^3^	0.2654	11.0^3^
Cr$_2$AlN	5.06	17.0	0.2975	**16.5**
Cr$_2$CuN	5.48	19.1	0.2876	**14.7**
Cr$_2$GeN	5.51	19.5	0.2822	**13.8**
Cr$_2$SnN	5.94	21.8	0.2730	**12.2**
Fe$_2$AlN	4.83	17.5	0.2764	**12.9**
Fe$_2$CuN	5.43	19.5	0.2791	**13.3**
Fe$_2$GeN	5.26	19.9	0.2641	**10.7**
Fe$_2$SnN	5.96	22.1	0.2696	**11.6**
Nb$_2$AlN	5.88	21.3	0.2761	**12.6**
Nb$_2$CuN	5.45	22.9	0.2375	**5.2**
Nb$_2$GeN	6.32	23.3	0.2707	**11.9**
Nb$_2$SnN	6.29	25.2	0.2492	**7.9**
Mo$_2$AlN	5.83	21.6	0.2701	**11.8**
Mo$_2$CuN	5.97	23.2	0.2572	**9.4**
Mo$_2$GeN	6.30	23.6	0.2669	**11.1**
Mo$_2$SnN	6.32	25.5	0.2480	**7.8**

The results in Fig. 3 show that for most M the data naturally fall into two classes (for X = C and X = N), and that our previously published Nb and Ti results are consistent with these new data, which reinforces the validity of the high-throughput model. The one exception seems to be the M$_2$SnX phases, which was also evident in the different ‘best fit’ value of $\mu ^{*}$ suggested by Fig. 1 and by the difference between experimental and model $T_c$ in Fig. 2. This may be due to different electronic hybridization with Sn but has not been investigated in detail. We also note that the $T_c$ value for Ti$_2$SnC reported in Table 1 has been calculated using Eliashberg theory, whereas in our previous paper^3^ it was predicted to be 7.1 K using the Fröhlich model. This is the largest difference that we could find between the model and Eliashberg theory. Hence, we are confident that the overall predictions of trends and $T_c$ are trustworthy. These results show that M = Cr has the highest $T_c$ as vary A and X, and that Cr$_2$AlN has the highest predicted $T_c$ = 16.5 K of any M$_2$AX phase studied so far.

Having identified Cr$_2$AlN as the most promising superconducting M$_2$AX phase, we now focus on a detailed study on its properties.

### Structural, electronic and Fermi surface properties of Cr$_2$AlN

**Figure 4:**
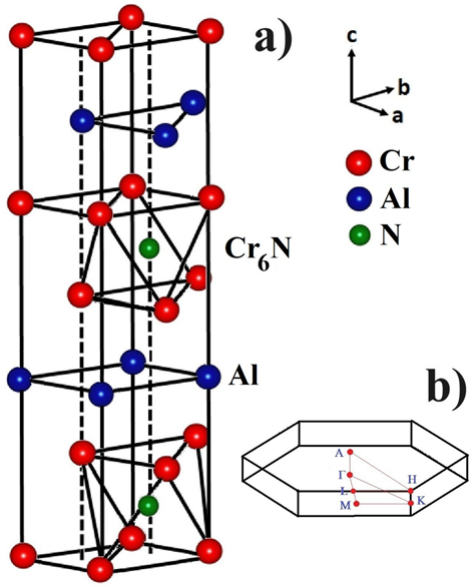
(**a**) The hexagonal crystal structure of Cr$_2$AlN, where blocks of Cr–N (formed by edge-shared Cr$_6$N octahedra) are sandwiched with Al atomic sheets. (**b**) The hexagonal Brillouin zone for Cr$_2$AlN.

To the best of our knowledge, Cr$_2$AlN has not yet been experimentally studied although there have been a few theoretical studies of its structural properties. Figure 4 shows the hexagonal unit cell for Cr$_2$AlN with space group P6$_3$/mmc. This is constituted of blocks of edge-sharing Cr$_6$N octahedra sandwiched by planes of Al. The primitive unit cell of Cr$_2$AlN include two formula units (eight atoms): four Cr atoms located at 4f (1/3, 2/3, *z*), two Al atoms at 2d (1/3, 2/3, 3/4), and two N atoms at (0, 0, 0). Thus, for Cr$_2$AlN, the structural information is completely determined by two lattice parameters (*a* and *c*) and one internal structural parameter (*z*). The optimised lattice constants (*a*, *c*), internal parameter(*z*), the closest Cr–Cr(d$_{Cr-Cr}$), Cr–Al (d$_{Cr-Al}$) Cr–N (d$_{Cr-N}$) and obtained the bulk modulus (*B*) and its pressure derivative ($B'$) of Cr$_2$AlN are shown Table 4. The computed values of *a* and *c* in Table 4 are remarkably close to the other theoretical values^32,33,54^. Table 4 also contains data for relaxed bond lengths of Cr$_2$AlN, where d$_{Cr-Cr}$, d$_{Cr-Al}$ and d$_{Cr-N}$ represent the bond length between appropriate pairs of atoms. The Cr–Cr distance is longer than in elemental Cr (2.498Å). The Cr–N bond-length of 1.942 Å, is almost equal to the sum of the covalent radii of Cr (1.22 Å) and N (0.70 Å), and is shorter than the Cr–Al distance suggesting that the Cr-N bond is stronger than the Cr–Al bond. We also performed various spin-polarized calculations and found that the relaxed ground state had negligible magnetization in each case, which agrees with a previous theoretical result^31^. Hence we proceed with assuming a non-magnetic ground state for Cr$_2$AlN.

**Table 4:** Structural properties of the hexagonal Cr$_2$AlN for this work in bold. Other theoretical results are also included for comparison.

Phase	*a* (Å)	*c* (Å)	z	d$_{Cr-Cr}$ (Å)	d$_{Cr-Al}$ (Å)	d$_{Cr-N}$ (Å)	B (GPa)	B$^{'}$
**Cr**$_2$ **AlN**	**2.832**	**12.664**	**0.083**	**2.658**	**2.677**	**1.942**	**175.10**	**1.59**
GGA^32^	2.839	12.708	0.082				158.07	4.48
GGA^33^	2.843	12.672			2.691	1.943		
GGA^54^	2.847	12.689	0.082				229	

**Figure 5:**
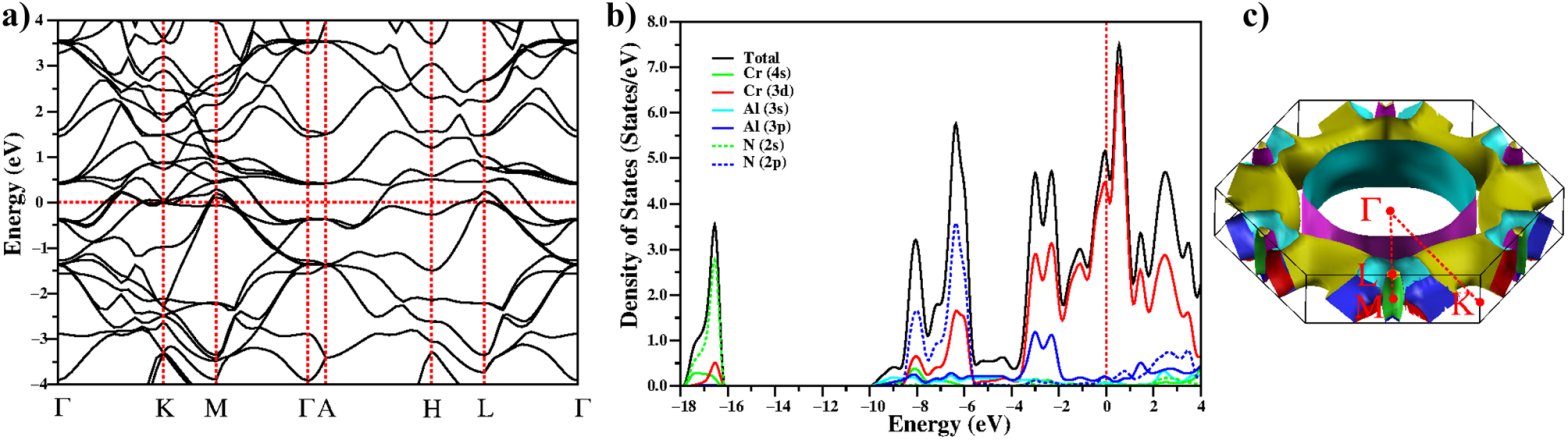
Electronic properties of the hexagonal phase of Cr$_2$AlN. (**a**) The electronic band structure, (**b**) the total and partial electronic local density of states, and (**c**) the Fermi surface.

Figure 5 shows the electronic characteristics of hexagonal Cr$_2$AlN, including (a) band structure in the Brillouin zone, (b) total and partial density of states (DOS and PDOS), and (c) the Fermi surface. Four dispersive bands cross at E$_F$, indicating that the electronic structure of this compound is multi-band and has metallic properties. The density of states at the Fermi level (N(E$_F$)) is crucial for determining metallic phases and superconductivity. Hence, the total and partial density of states for Cr$_2$AlN are shown in Fig. 5b. For Cr$_2$AlN, the lowest energy region, ${-17.9<E<-16.2}$ eV, is dominated mainly by the N 2s states with small contributions from Cr 4s and 3d states. The valence band is mainly composed of ${-10.0< E <-5.5 }$ eV states that come from Cr 3d and N 2p. In this valence region, Cr and N atoms exhibit hybridization, indicating the possibility of a covalent bond between these elements. The main region of the valence band, which extends between ${-3.8< E < -1.8}$ eV, is formed of hybridised states of Cr 3d and Al 3p, which indicates the existence of covalent Cr–Al bonding. The Cr–N bonds represent a lower energy range than the Cr–Al bonds, indicating that the Cr–N bonds have a higher binding energy and are stronger than the Cr–Al bonds, which is comparable to the findings obtained when analyzing bond distance. The DOS of Cr$_2$AlN has peaks with a lower energy than the other M$_2$AX phases we have calculated^2,3^ and it is known that hybridized states with lower energy lead to stronger bonding among M$_2$AX phases^55,56^. Thus, the DOS peaks of Cr$_2$AlN may suggest that the bonding between atomic states in this compound is stronger than in the other M$_2$AX phases which could help explain the high $T_c$.

The value of N(E$_F$) is proportional to the electron–phonon coupling parameter ($\lambda$) according to the McMillan–Hopfield expression^40^:

9 $$\begin{aligned} \lambda =\frac{N(E_{F}){\langle {I^2}\rangle }}{M{\langle {\omega ^2}\rangle }}, \end{aligned}$$

where ${\langle {\omega ^2}\rangle }$ denotes the average squared phonon frequency, ${\langle {I^2}\rangle }$ describes the average squared electron–phonon matrix element and M is the average atomic mass. As Cooper pairs of electrons having energies close to the Fermi level, the electronic density of states at the Fermi energy plays a crucial role in superconductivity, and we find N(E$_F$)=5.06 states/eV for Cr$_2$AlN, which is a high value when compared to similar structures^2,3^. Figure 5b shows that the peak at the Fermi level is dominated by Cr 3d orbitals, indicating that the 3d electrons dominate the conduction properties. The contributions of Cr, Al, and N atoms to DOS are 89%, 6%, and 5%, respectively. As a result, the electronic states from Cr are responsible for the conducting and superconducting properties of Cr$_2$AlN, and as this has the highest value of $N(E_F)/\sqrt{M}$ among the M$_2$AX phases studied, it should (if all other effects are similar), have the highest $T_c$ value.

Figure 5c shows a plot of the Cr$_2$AlN Fermi surface, which is quite complex. The electrical conductivity of Cr$_2$AlN is formed mainly by the Cr 3d-like bands on the Fermi surface, which contains six different sheets. Hole-like sheets appear in the corners of the Brillouin zone along the *H*-*K* and *L*–*M* directions, while the Fermi surface is completely prismatic and cylindrical in the $\Gamma$-*A* direction and shows electron-like behaviour. The first sheet of Cr$_2$AlN is cylindrical whilst other sheets interact with each another. The Fermi surface of Cr$_2$AlN is similar to the other M$_2$AX phase Fermi surfaces in our previous papers^2,3^.

### Elastic and mechanical properties of Cr$_2$AlN

The elastic properties of solids are a link between mechanical and vibrational properties. In the phonon-mediated version of the BCS theory, phonons couple electrons to form Cooper pairs which are the foundation of superconductivity. In this study, elastic constant calculations have been done using the strain–stress method as performed in the thermo_pw package^57^: a set of small strains is applied to the crystal lattice and then the lattice containing the atoms are totally relaxed to obtain the corresponding stress. The symmetry of the hexagonal lattice results in five independent elastic constants: C$_{11}$, C$_{12}$, C$_{13}$, C$_{33}$ and C$_{44}$, which are given in Table 5. As shown in Table 5, C$_{33}$ greater than C$_{11}$, indicating that Cr$_2$AlN is more incompressible along the z-axis than along the x-axis. In other words, the bonding strength along the z-axis is significantly greater than along the x-axis. The mechanical stability criteria determined by the Born Huang lattice dynamics theory^58,59^ can be given as: C$_{11}$ $|C_{12}|$; (C$_{11}$+2C$_{12}$)C$_{33}$-2C${_{13}^2}$ 0 and C$_{44}$ 0. It is clear from Table 5 that Cr$_2$AlN elastic constants meet the mechanical stability criteria.

**Table 5:** The calculated second order elastic constants (in GPa) for hexagonal superconductor Cr$_2$AlN for this work in bold. Other theoretical results are also included for comparison.

Phase	C$_{11}$	C$_{12}$	C$_{13}$	C$_{33}$	C$_{44}$
**Cr**$_2$ **AlN**	**286.66**	**83.43**	**147.81**	**378.35**	**78.84**
GGA^32^	282	85	141	362	77
GGA^33^	279.52	81.90	143.57	370.31	77.65
GGA^34^	286.67	74.85	144.539	376.67	88.24

Using the well-known Voigt–Reuss–Hill (VRH)^60–62^ approximations, it is possible to calculate the isotropic bulk modulus ($B_{VRH}$), the isotropic shear modulus ($G_{VRH}$), Young’s modulus (E), and Poisson’s ratio ($\sigma$) from the elastic constants. The general Voigt–Reuss–Hill formulas for hexagonal crystals are given by the following equations:

10 $$\begin{aligned} B_V= & {} \frac{2}{9}(C_{11}+C_{12}+2C_{13}+C_{33}/2), \end{aligned}$$

11 $$\begin{aligned} G_V= & {} \frac{1}{15}(2C_{11}+C_{33}-C_{12}-2C_{13})+\frac{1}{5}(2C_{44}+C_{66}), \end{aligned}$$

12 $$\begin{aligned} B_R= & {} \frac{1}{(2S_{11}+S_{33})+2(S_{12}+2S_{13})}, \end{aligned}$$

13 $$\begin{aligned} G_R= & {} \frac{15}{4(2S_{11}+S_{33})-4(S_{12}+2S_{13})+3(2S_{44}+S_{66})} \end{aligned}$$.

14 $$\begin{aligned} B_H= & {} \frac{B_V+B_R}{2}, \end{aligned}$$

15 $$\begin{aligned} G_H= & {} \frac{G_V+G_R}{2}, \end{aligned}$$

16 $$\begin{aligned} E= & {} \frac{9B_HG_H}{(3B_H+G_H)}, \end{aligned}$$

17 $$\begin{aligned} \sigma= & {} \frac{3B_H-2G_H}{2(3B_H+G_H)}, \end{aligned}$$

where $S_{ij}$ is the elastic compliance constants. The calculated values of bulk modulus ($B_V$, $B_R$ and $B_H$), shear modulus ($G_V$, $G_R$ and $G_H$), Young’s modulus (*E*), the ratio of bulk to shear modulus ($B_H$/$G_H$) and Poisson’s ratio ($\sigma$) are shown in Table 6. The calculated values agree with other theoretical values^32–34^ and the values of $B_H$ are close to *B* determined by total energy calculations in Table 4.

**Table 6:** The estimated values of isotropic bulk modulus B$_{VRH}$, shear modulus G$_{VRH}$, Young’s modulus E (all in GPa), B$_H$/G$_H$ ratio and Poisson’s ratio ($\sigma$) for Cr$_2$AlN for this work in bold, obtained from their second order elastic constants C$_{ij}$. Other theoretical results are also included for comparison.

Phase	B$_V$	B$_R$	B$_H$	G$_V$	G$_R$	G$_H$	E	B$_H$/G$_H$	$\sigma$
**Cr**$_2$ **AlN**	**189.97**	**179.63**	**184.80**	**90.03**	**87.88**	**88.96**	**229.96**	**2.077**	**0.293**
GGA^32^		177	181	88	86	87		2.10	
GGA^33^			187.00			136.12	328.62	0.728	0.207
GGA^34^	186.43	175.872	181.15	95.55	93.63	94.59	241.697	1.915	0.278

The ratio of bulk modulus to shear modulus ($B_H$/$G_H$) can be used with the empirical formula of Pugh^63^ to indicate that a material is ductile (brittle) if $B_H$/$G_H$ is greater (smaller) than 1.75. The calculated value of $B_H$/$G_H$ for Cr$_2$AlN is 2.077 which suggests Cr$_2$AlN should be ductile, which is favorable for its technological applications. Moreover, the Poisson’s ratio of Cr$_2$AlN is $\nu =0.293$. This is greater than the ductile critical value^64^ of 0.26. It is known that $\nu \approx 0.1$ in covalent and $\nu \approx 0.33$ in metallic materials^64^ and so as Cr$_2$AlN has $\nu =0.293$, it should have metallic behavior.

### Phonons and electron–phonon interaction of Cr$_2$AlN

**Figure 6:**
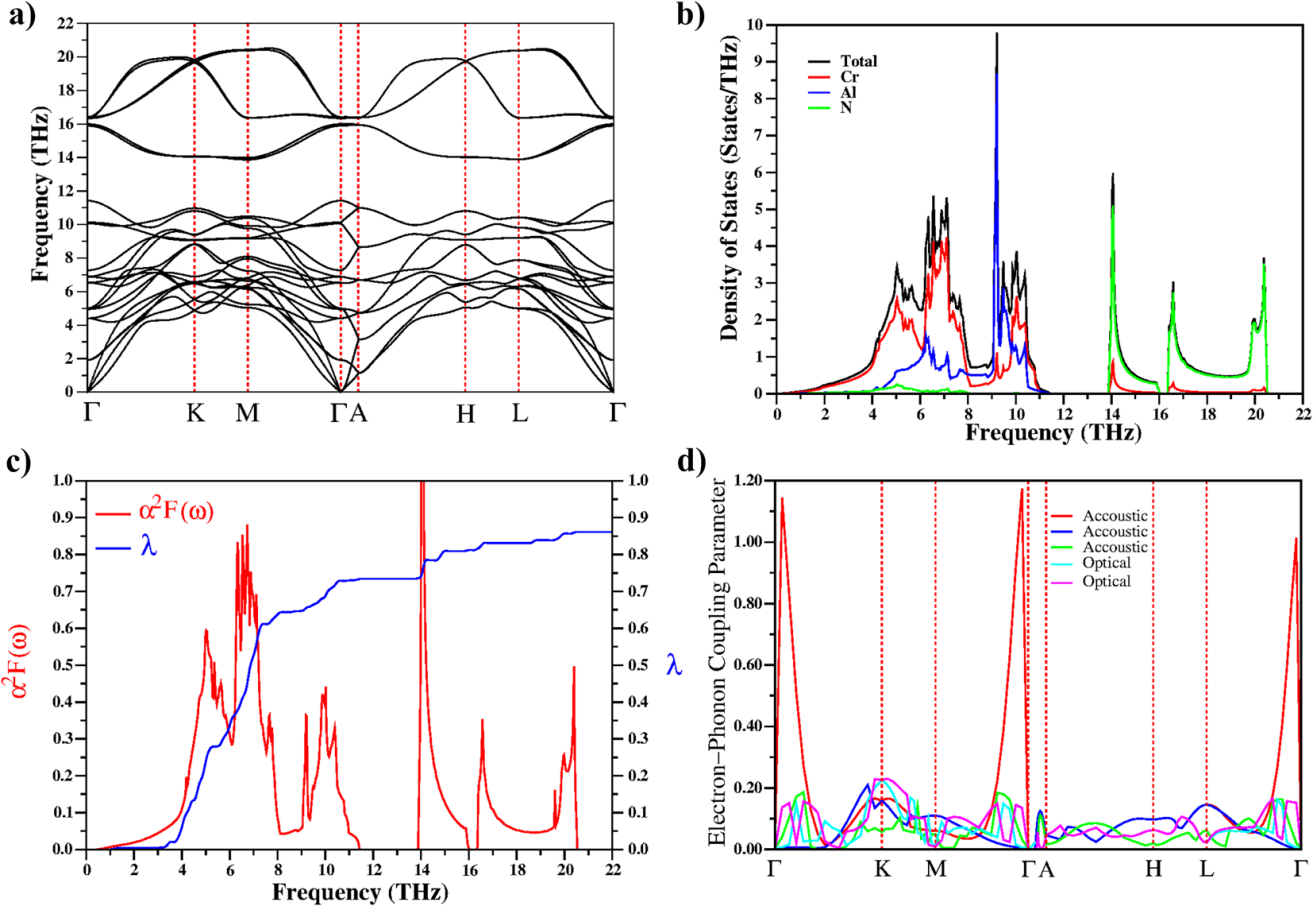
The calculated phonon properties of Cr$_2$AlN. (**a**) dispersion curves, (**b**) total and partial phonon density of states, and (**c**) the calculated electron–phonon spectral function $\alpha ^2 F(\omega )$ (red line) and the variation of the electron–phonon coupling parameter (blue line) with rising frequency $\lambda$($\omega$). (**d**) Calculated wavevector-dependent electron–phonon coupling parameter for the 3 acoustic modes and 2 optical modes along the high symmetry lines in the Brillouin zone.

Phonon dispersion, total and partial vibrational density of states (VDOS) and electron–phonon spectral function for Cr$_2$AlN have been analyzed in detail because they play a crucial role in superconductivity. As the primitive unit cell of Cr$_2$AlN consists of eight atoms, there are 24 phonon modes, including 3 acoustic modes and 21 optical modes. Our previous paper on Nb-based materials has analysed the zone-centre optical phonon modes in hexagonal M$_2$AX phases in detail^2^. The zone-centre optical phonon modes of Cr$_2$AlN can be classified by the irreducible representations of the point group D$_{6h}$(6/mmm). The electron–phonon coupling parameter of the $A_{1g}$ phonon mode is significantly larger than the corresponding values for the other phonon modes at $\Gamma$ point. Figure 6 shows the calculated phonon dispersion relations (a), total and partial vibrational density of states (VDOS) (b), and electron-phonon spectral function for Cr$_2$AlN (c). Cr$_2$AlN is dynamically stable as there are no negative frequencies. Generally, the phonon properties of Cr$_2$AlN are very similar to the MAX phases in our previous studies^2,3^. The Cr$_2$AlN phonon spectrum can be separated into three apparent regions by two phonon band gaps of width 2.5 and 0.4 THz. The differences in the masses of the three atomic species cause these separations. The first phonon region, between 0 and 11.4 THz, contains three acoustic phonon modes and fifteen optical phonon modes. In order to define the nature of all phonon branches in the phonon dispersion diagram of Cr$_2$AlN, we present its total and partial phonon density of states in Fig. 6b. There is significant overlap and hybridization of Cr–Al modes in the low frequency region for Cr$_2$AlN (see Fig. 6b). The phonon modes up to 8 THz are dominated Cr atoms due to their heavier mass. The Cr and Al atoms vibrate and hybridize more strongly between 8 THz and 11.4 THz. There is no N atom contribution in this region. In the other two regions, the VDOS is dominated by the motions of the low mass N atoms, with negligible contribution from Cr.

One of the main aims of this study is to identify the electron orbitals and phonon modes that are most effective in developing superconductivity. For this reason, the Eliashberg spectral function ($\alpha ^2F(\omega )$) and the frequency variation of the average electron-phonon coupling parameter $\lambda$ for Cr$_2$AlN are presented in Fig. 6c. In our previous publications, we analysed the Eliashberg spectral function in detail^2,3^. Here, we find for Cr$_2$AlN that $\lambda$ = 0.861, and it increases strongly with frequency between 4 THz and 8 THz. An analysis of the Eliashberg spectral function reveals that the phonon modes with low frequency (the first phonon region, below 11.4 THz) contribute 85% to the total ($\lambda$ = 0.734), with modes dominated by the coupled motion of Cr and Al ions. This large contribution shows that these phonon modes couple strongly with electrons at the Fermi level. This strong coupling is to be expected as the corresponding phonon modes are dominated by Cr atoms, which dominate the electronic states close to the Fermi level. As the high-frequency region is dominated by light N atom modes, its contribution to $\lambda$ is minor. Among all the M$_2$AX phases we have studied, Cr$_2$AlN has the highest $\lambda$ value and hence might be expected to have the highest $T_c$.

The electron–phonon coupling parameter of the 3 acoustic modes and 2 optical modes along the high symmetry lines in the Brillouin zone are given Fig. 6d using Eq. 3. The electron–phonon coupling value near the $\Gamma$ point is particularly strong for the longitudinal acoustic mode along the $\Gamma$-K, M-$\Gamma$, and L-$\Gamma$ directions. Figure 5c shows that along these directions, there are a number of near-parallel sheets of the Fermi surface. These sheets can be coupled by a similar **q**-vector, an effect known as Fermi surface nesting. The large region of the Fermi surface coupled in this way will result in a strong electron–phonon interaction. Fermi nesting could be important in improving electron–phonon interactions^65,66^ and thus increasing superconductivity.

Using the value of $\lambda$, we calculate the logarithmic average phonon frequency for Cr$_2$AlN to be $\omega _{\ln }$=331.225 K. These values are used to calculate $T_c$ by using the Allen–Dynes modification of the McMillan equation as in Eq. 7. The value of $\mu ^{*}$ in most studies ranges from 0.10 to 0.16^40,41^. Here, as discussed above, we use $\mu ^{*}$=0.13 as it gave the best overall fit to the experimental $T_c$ as can be seen in Fig. 1. As there are no known experimental $T_c$ values for Cr$_2$AlN, we again use $\mu ^{*}$=0.13 to calculate $T_c$=14.814 K using the Eliashberg theory. Whilst this is a little lower than the value predicted by our Fröhlich model (developed on the basis of $\mu ^{*}$=0.13), it is considerably higher than the highest $T_c$ value for any material in the M$_2$AX family studied to date.

## Conclusion

We have used a high-throughput method to examine the superconducting properties of 53 new M$_2$AX phases (from M: Sc, Ti, V, Cr, Mn, Fe, Y, Zr, Nb, Mo, Lu, Hf, and Ta; A: Al, Ge, Cu and Sn; X: C and N) as given in Tables 1, 2, 3 and shown in Figs. 2 and 3. Of the materials studied, only four were previously known from experiment to be superconductors, and some had been predicted to be superconducting in our earlier works. According to our screening method, for each A (A: Al, Ge, Cu and Sn) the highest superconducting $T_c$ result is obtained with M = Cr and X = N. Whilst Nb$_2$GeC has the highest known experimental $T_c$ = 10 K^30^, the result of our high-throughput screening suggests that Cr$_2$AlN should have the highest $T_c$ = 16.5 K of any M$_2$AX phase. This model prediction is then tested with a more detailed electron–phonon coupling calculation using Eliashberg theory.

Our DFT calculations show that Cr$_2$AlN is mechanically and dynamically stable, with ductile and metallic properties. The electron–phonon interaction is dominated by low-frequency Cr-based phonon modes and Cr 3d-based electronic states near the Fermi energy. Using Eliashberg theory, we calculate the total $\lambda$ = 0.861 and hence using the most likely value of $\mu ^{*}$ = 0.13, we predict $T_c$ = 14.8 K for Cr$_2$AlN. Based on our previous experience of comparing Eliashberg theory for M$_2$AX phases with experiment, we expect this to be within ± 1 K of experiment. This value is considerably higher than the highest $T_c$ value for any material in the M$_2$AX family studied to date. This finding should encourage further study into the superconductivity of M$_2$AX phases in general, and of Cr$_2$AlN in particular.

## Supplementary information

In this paper we have presented detailed data on the electronic structure, phonons and electron–phonon coupling calculations for Cr$_2$AlN. As part of this study, we also used Migdal–Eliashberg (ME) theory to study 8 other materials, whose data is summarized in Table 1. The ME data for Nb$_2$GeC and Nb$_2$SnC have already been presented in Ref.^2^ although the final $T_c$ value was calculated with $\mu ^{*}$ = 0.10. The ME data for Ti$_2$GeC have already been presented in Ref.^3^. Hence we include the full data needed for the ME calculations on the other 5 materials (Mo$_2$GeC, Lu$_2$GeC, Ti$_2$SnC, Mo$_2$SnC and Lu$_2$SnC) in the Supplementary Information to this paper.

## Supplementary Information

Supplementary Information.

## Data Availability

The data created and analysed during the current study are available from https://doi.org/10.15124/85422e07-e03d-49f9-81e1-eccea2943796.
