# Supplementary material for: Cr2AlN and the search for the highest temperature superconductor in the M2AX family
Source: Sci Rep. 2023 Apr 21;13:6576. doi: 10.1038/s41598-023-33517-0 (PMC10121671; doi:10.1038/s41598-023-33517-0)
Supplement: Supplementary file 1 — Supplementary Information. [file 41598_2023_33517_MOESM1_ESM.pdf]

Supplementary Material: Cr_2AlN And The Search For The Highest Temperature Superconductor In The M_2AX Family

Karaca, E.[1,2]; Byrne, P. J. P.[1]; Hasnip, P.J.[1] and Probert, M.I.J.[1]
[1] *Department of Physics, University of York,*
York YO10 5DD, United Kingdom
and
[2] *Sakarya University, Biomedical,*
Magnetic and Semiconductor Materials Research Center (BIMAS-RC), 54187, Sakarya, Turkey

A. Structural, electronic and Fermi surface properties of M_2AC (M: Ti, Mo and Lu; A: Ge and Sn) compounds

The structural, electronic, Fermi surface, phonon, and electron-phonon properties of M_2GeC (M: Mo and Lu) and M_2SnC (M: Ti, Mo and Lu) have been examined by *ab initio* pseudopotential calculations within the generalized gradient approximation (GGA) of the density functional theory (DFT). Of these, only Lu_2SnC was previously known to be superconducting in experiment. We have recently performed theoretical studies of superconductivity in Ti_2GeC, Nb_2GeC, and Nb_2SnC[1, 2]. In the previous paper[1], μ^* for Nb_2GeC and Nb_2SnC was set at 0.10, which was determined by matching the Fröhlich model to a series of Nb-based MAX phases; when studying a wider range of MAX-phase materials[2] and this work, it was found that μ^*=0.13 was a better fit, and hence this latter value was used throughout this study. This change causes a small decrease in the predicted T_c for Nb_2GeC and Nb_2SnC compared to ref. [1] but all other results are the same. Hence in this SI we will focus on the 5 materials which have been studied here using the full Eliashberg approach and whose results have not previously been reported.

There are two formula units and eight atoms in a primitive cell for these crystals, and they crystallise in the hexagonal structure ($P6_3/mmc$), as shown in Figure S1 (repeated from figure 2 in main text). The crystal structure properties of Cr_2AlN are discussed in the main text. Here we give the properties of M_2GeC(M: Mo and Lu) and M_2SnC(M: Ti, Mo and Lu). The calculated equilibrium lattice constants (a,c), internal parameter (z), bulk modulus, and its pressure derivative are listed in Table S1, together with experimental data and previous theoretical results. The calculated results are in good agreement with previous experimental [3–5] and theoretical[6, 7] although there have been no publications on Lu_2GeC material to yet.

The electronic band structure, the total and partial density of states, and the Fermi surface of M_2GeC(M: Mo and Lu) and M_2SnC(M: Ti, Mo and Lu) are presented in figure S2 and figure S4, respectively. It can be seen from the figures that all of them have metallic properties, with the density of states at the Fermi energy N(E_F) is dominated by the M(M: Ti, Mo and Lu) d states. The calculated values for N(E_F) are listed in table S2. Also, Ge and Sn p states in Lu_2GeC and Lu_2SnC materials significantly contribute to N(E_F).

The Fermi surfaces of M_2GeC (M: Mo and Lu) and M_2SnC (M: Ti, Mo and Lu) are roughly similar to those of Cr_2AlN, which is discussed in the main text. The calculated Fermi surfaces agree with previous theoretical results[8–10]. The properties of M_2GeC(M: Mo and Lu) and M_2SnC(M: Ti, Mo and Lu) are mostly set by the d orbitals of the M (M:Ti, Mo and Lu) atoms at the Fermi level N(E_F). The Fermi surface of Ti_2SnC has six Fermi surface sheets, whilst Mo_2GeC and Mo_2SnC have 5 and Lu_2GeC and Lu_2SnC have 4. As the mass of the M atom increases, the number of bands that cross the Fermi levels tends to decrease, reducint N(E_F). As with Cr_2AlN, hole-like sheets occur in the corners of the Brillouin zone in the H-K and L-M directions, whilst the Fermi surface is completely prismatic and cylindrical in the Γ-A direction and exhibits electron-like behaviour. We also see that Mo_2GeC and Mo_2SnC

materials exhibit strong Fermi nesting, similar to that of Cr_2AlN, which can increase electron-phonon interaction.

B. Phonon and electron-phonon interaction of M_2AC (M: Ti, Mo and Lu; A: Ge and Sn) compounds

The calculated phonon spectrum, total and partial vibrational density of states (VDOS), and electron–phonon spectral function are presented in figure S3 and figure S5. All of them are dynamically stable as only positive phonon modes are exist. Lu_2GeC and Lu_2SnC has 2 distinct frequency regions: a low-frequency area up to 7 THz with three acoustic and fifteen optical phonon modes, and a high-frequency region between 11 and 17 THz with six optical modes. These two regions are separated by a large gap. Both the high-frequency and low-frequency regions of Lu_2GeC are divided into two regions by small gaps. The phonon band structure of all other materials can be divided into three distinct regions. For M_2GeC (M: Mo and Lu) and M_2SnC (M: Ti, Mo and Lu), there is overlap and hybridization of M - A modes in the low-frequency region. The high frequency region has two peaks separated by a small gap for Mo_2GeC, M_2SnC(M:Ti or Mo) and Lu_2GeC, but just a single peak for Lu_2SnC.

The frequency dependence of the Eliashberg spectral function ($\alpha^2F(\omega)$) and the electron-phonon interaction parameter (λ) are shown in figure S3 and figure S5, respectively, and can be used to investigate the impact of electron-phonon interaction on the superconducting properties of M_2GeC(M: Mo and Lu) and M_2SnC(M: Ti, Mo and Lu). The spectral function ($\alpha^2F(\omega)$) significantly contributes to the vibrational modes at low frequencies. Then, using the evaluated values of λ, the reduced Coulomb parameter (μ^*=0.13) and $\omega_{\ln}$ in the Allen-Dynes modified McMillan formula, the value of T_c of Mo_2GeC, Lu_2GeC, Ti_2SnC, Mo_2SnC and Lu_2SnC is estimated to be 9.607, 2.504, 5.897, 7.635 and 5.963, respectively. The calculated T_c value of Lu_2SnC agrees well with the known experimental result[5].

---

[1] E. Karaca, P. J. P. Byrne, P. J. Hasnip, H. Tütüncü, and M. Probert, Electron–phonon interaction and superconductivity in hexagonal ternary carbides Nb_2AC (A: Al, S, Ge, As and Sn), Electronic Structure **3**, 045001 (2021).

[2] E. Karaca, P. J. P. Byrne, P. J. Hasnip, and M. Probert, Prediction of phonon-mediated superconductivity in new Ti-based M_2AX phases, Scientific reports **12**, 13198 (2022).

[3] Z. Du, S. Wang, R. Hu, D. Zhang, J. Gu, X. Chen, J. Shang, B. Li, S. Yang, and L. Guo, Unlocking the catalytic activities of 2H-phase Mo-based compounds via topological conversion reaction, Materials Today **51**, 136 (2021).

[4] M. Barsoum, G. Yaroschuk, and S. Tyagi, Fabrication and characterization of M_2SnC (M= Ti, Zr, Hf and Nb), Scripta Materialia **37**, 1583 (1997).

[5] S. Kuchida, T. Muranaka, K. Kawashima, K. Inoue, M. Yoshikawa, and J. Akimitsu, Superconductivity in Lu_2SnC, Physica C: Superconductivity **494**, 77 (2013).

[6] M. Cover, O. Warschkow, M. Bilek, and D. McKenzie, A comprehensive survey of M_2AX phase elastic properties, Journal of Physics: Condensed Matter **21**, 305403 (2009).

[7] M. M. Ali, M. Hadi, I. Ahmed, A. Haider, and A. Islam, Physical properties of a novel boron-based ternary compound Ti_2InB_2, Materials Today Communications **25**, 101600 (2020).

[8] M. Hadi, N. Kelaidis, S. Naqib, A. Chroneos, and A. Islam, Electronic structures, bonding natures and defect processes in Sn-based 211 MAX phases, Computational Materials Science **168**, 203 (2019).

[9] D. Pinek, T. Ito, K. Furuta, A. J. van Bunningen, P. Le Fèvre, F. Bertran, and T. Ouisse, Fermi surface and band structure of Ti_2SnC as observed by angle-resolved photoemission spectroscopy, Physical Review B **104**, 195118 (2021).

[10] M. Kanoun, S. Goumri-Said, and A. H. Reshak, Theoretical study of mechanical, electronic, chemical bonding and optical properties of Ti_2SnC, Zr_2SnC, Hf_2SnC and Nb_2SnC, Computational Materials Science **47**, 491 (2009).

[11] M. Khazaei, M. Arai, T. Sasaki, M. Estili, and Y. Sakka, The effect of the interlayer element on the exfoliation of layered Mo_2AC (A= Al, Si, P, Ga, Ge, As or In) MAX phases into two-dimensional Mo2C nanosheets, Science and technology of advanced materials **15**, 014208 (2014).

[12] D. Huang, R. Qiu, C. Mo, and T. Fa, Structural, mechanical, dynamical and electronic properties and high-pressure behavior of Mo_2GeC: a first-principles study, Computational Materials Science **137**, 306 (2017).

[13] Y. Shao, D. Zhao, and W. Duan, First-principles investigation of the intrinsic defect-related properties in Mo_2GeC, AIP Advances **10**, 025136 (2020).

[14] A. Bouhemadou, Calculated structural, electronic and elastic properties of M_2GeC (M= Ti, V, Cr, Zr, Nb, Mo, Hf, Ta and W), Applied Physics A **96**, 959 (2009).

[15] H. Vincent, C. Vincent, B. Mentzen, S. Pastor, and J. Bouix, Chemical interaction between carbon and titanium dissolved in liquid tin: crystal structure and reactivity of Ti_2SnC with Al, Materials Science and Engineering: A **256**, 83 (1998).

[16] C. Lu, Y. Liu, J. Fang, Y. Zhang, P. Zhang, and Z. Sun, Isotope study reveals atomic motion mechanism for the formation of metal whiskers in MAX phase, Acta Materialia **203**, 116475 (2021).

[17] G. Hug, Electronic structures of and composition gaps among the ternary carbides Ti_2MC, Physical Review B **74**, 184113 (2006).

[18] A. Bouhemadou, Prediction study of structural and elastic properties under pressure effect of M_2SnC (M= Ti, Zr, Nb, Hf), Physica B: Condensed Matter **403**, 2707 (2008).

[19] M. Hadi, N. Kelaidis, S. Naqib, A. Chroneos, and A. Islam, Mechanical behaviors, lattice thermal conductivity and vibrational properties of a new MAX phase Lu_2SnC, Journal of Physics and Chemistry of Solids **129**, 162 (2019).

[20] Y. Shao and W. Duan, First-principles study of mechanical and electronic properties of Lu_2SnC under pressure, Journal of Applied Physics **127**, 155902 (2020).

[21] M. Hadi, S.-R. Christopoulos, A. Chroneos, S. Naqib, and A. Islam, Dft insights into the electronic structure, mechanical behaviour, lattice dynamics and defect processes in the first sc-based max phase sc2snc, Scientific reports **12**, 14037 (2022).

TABLE S1. Structural properties of M_2GeC (M: Mo and Lu) and M_2SnC (M: Ti, Mo and Lu) for this work in bold, and their comparison with previous experimental and theoretical results.

Phase	a(Å)	c(Å)	z	d_{M-M}(Å)	d_{M-A}(Å)	d_{M-C}(Å)	B(GPa)	B'
**Mo_2GeC**	**3.131**	**12.632**	**0.089**	**2.888**	**2.719**	**2.129**	**216.3**	**4.67**
Exp[3]	3.022							
GGA[6]	3.149	12.640					212	
GGA[11]	3.135	12.635	0.089		2.718	2.134	214.4	
GGA[12]	3.130	12.653	0.089		2.720	2.130	210	4.75
GGA[13]	3.120	12.609						
LDA[14]	3.121	12.332	0.092				225	4.55
**Lu_2GeC**	**3.482**	**14.577**	**0.089**	**3.518**	**3.291**	**2.475**	**92.6**	**4.11**
**Ti_2SnC**	**3.176**	**12.610**	**0.081**	**2.737**	**2.816**	**2.096**	**136.9**	**4.14**
Exp[4]	3.163	13.675						
Exp[15]	3.163	13.679						
Exp[16]	3.180	13.860						
GGA[7]	3.174	13.781						
GGA[10]	3.136	13.641	0.082		2.923	2.126	170.0	
GGA[17]	3.171	13.857	0.081				167.0	
GGA[18]	3.096	13.404	0.083				159.3	4.29
**Mo_2SnC**	**3.191**	**12.409**	**0.080**	**2.717**	**2.796**	**2.095**	**187.7**	**5.35**
GGA[6]	3.203	13.520					184	
**Lu_2SnC**	**3.514**	**15.377**	**0.083**	**3.268**	**3.269**	**2.399**	**89.1**	**4.51**
Exp[5]	3.514	15.159						
GGA[19]	3.546	15.323	0.085					
GGA[20]	3.542	15.315						
GGA[21]	3.546	15.323	0.085	3.314	3.255	2.428	86	

TABLE S2. Density of states at the Fermi level ($N(E_F)$), logarithmic frequency ($\omega_{\ln}$), the average electron-phonon coupling parameter (λ) and the superconducting transition temperature (T_c in K) for the hexagonal M_2GeC (M: Mo and Lu) and M_2SnC (M: Ti, Mo and Lu) for this work in bold, and their comparison with available previous experimental and theoretical results.

Phase	$N(E_F)$(states/eV)	$\omega_{\ln}$ (K)	λ	T_c (K)
**Mo_2GeC**	**4.125**	**205.320**	**0.879**	**9.607**
**Lu_2GeC**	**4.372**	**188.675**	**0.402**	**2.504**
**Ti_2SnC**	**3.427**	**362.703**	**0.602**	**5.897**
GGA[7]	3.360			
GGA[10]	3.300			
GGA[17]	3.710			
**Mo_2SnC**	**4.838**	**218.861**	**0.774**	**7.635**
**Lu_2SnC**	**5.177**	**105.345**	**0.798**	**5.963**
Exp[5]				5.2

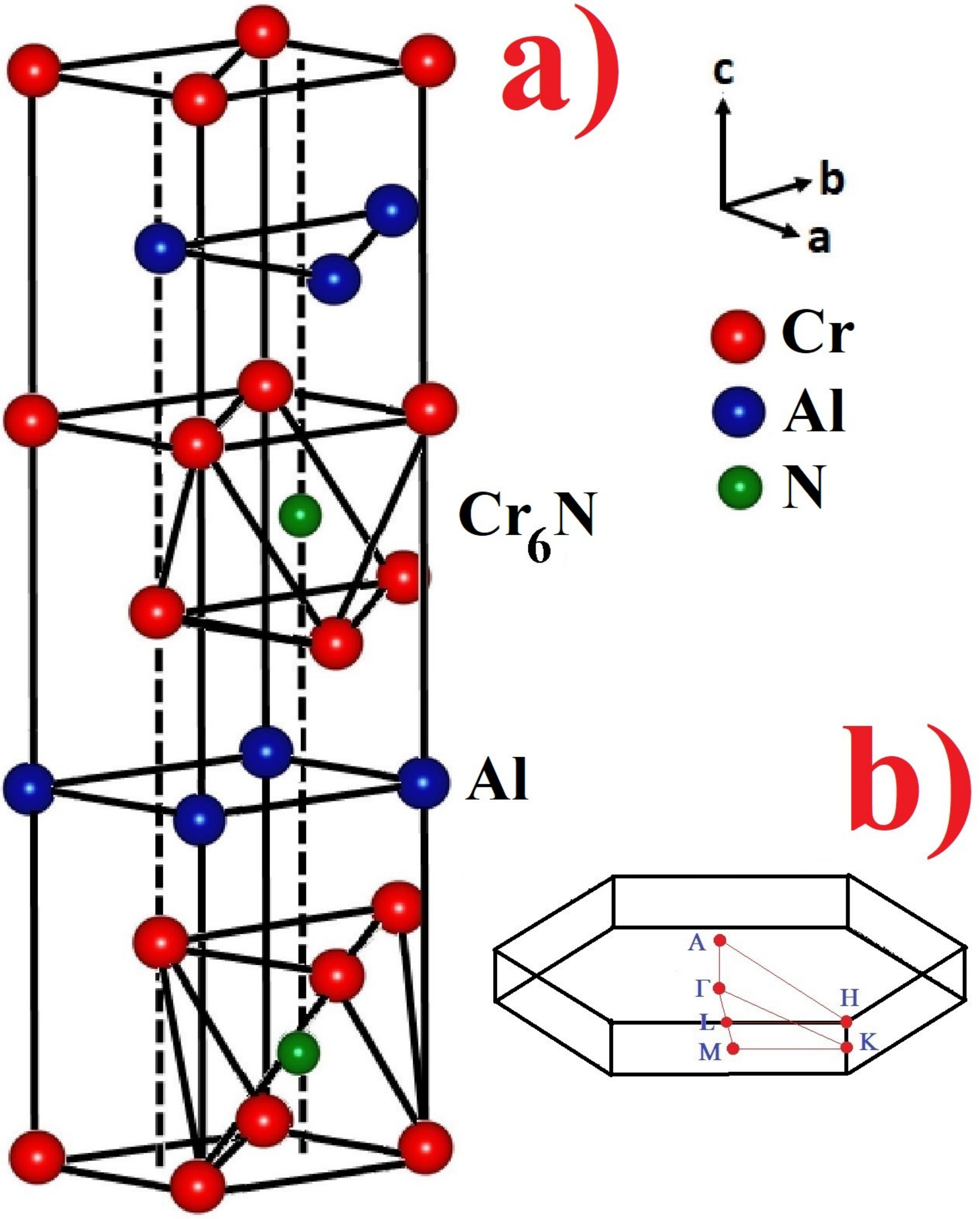

FIG. S1. (a) The hexagonal crystal structure of Cr_2AlN, where blocks of Cr-N (formed by edge-shared Cr_6N octahedra) are sandwiched with Al atomic sheets. (b) The hexagonal Brillouin zone for Cr_2AlN.

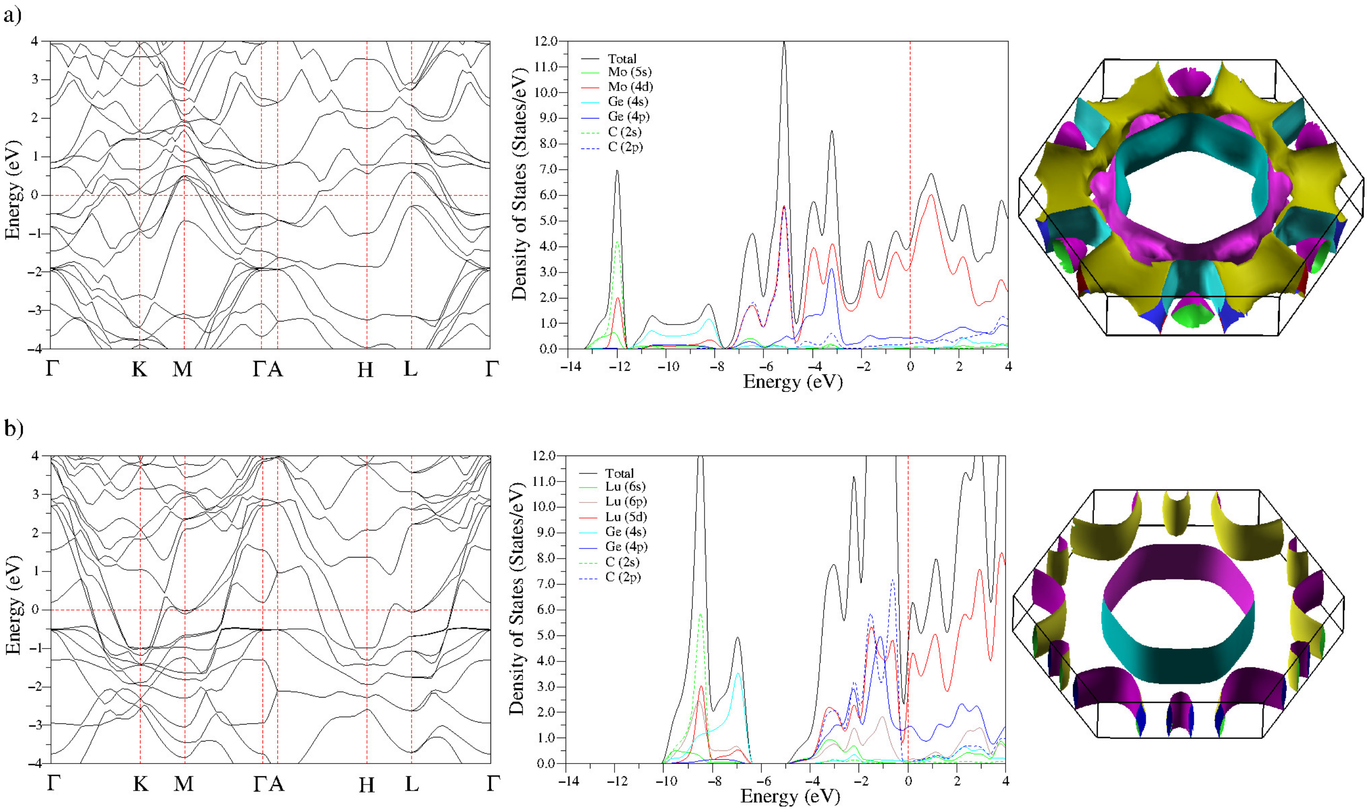

FIG. S2. The electronic band structure, the total and atomic projected electronic local density of states and Fermi surface for the hexagonal phase of M_2GeC (M: Mo and Lu).

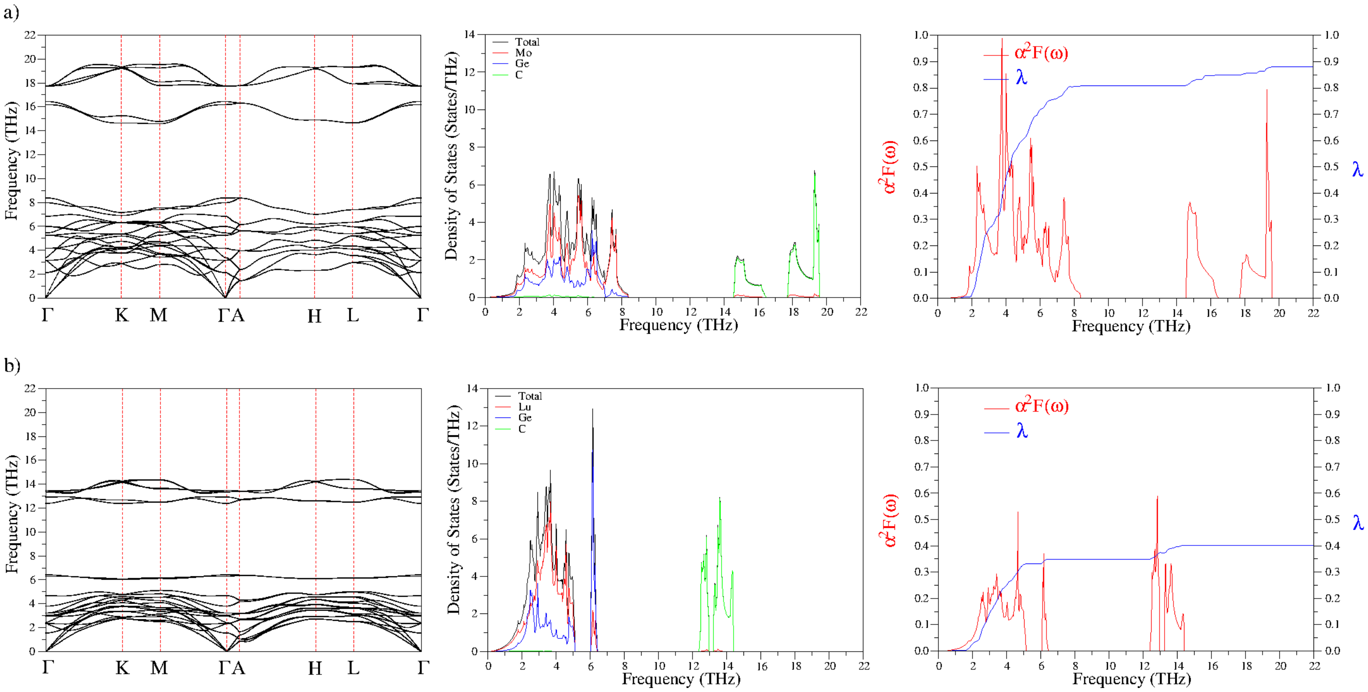

FIG. S3. Phonon dispersion curves, total, partial vibrational density of states and the calculated electron–phonon spectral function $\alpha^2 F(\omega)$ (red line) and the variation of the electron–phonon coupling parameter (blue line) with rising frequency $\lambda(\omega)$ of M_2GeC (M: Mo and Lu).

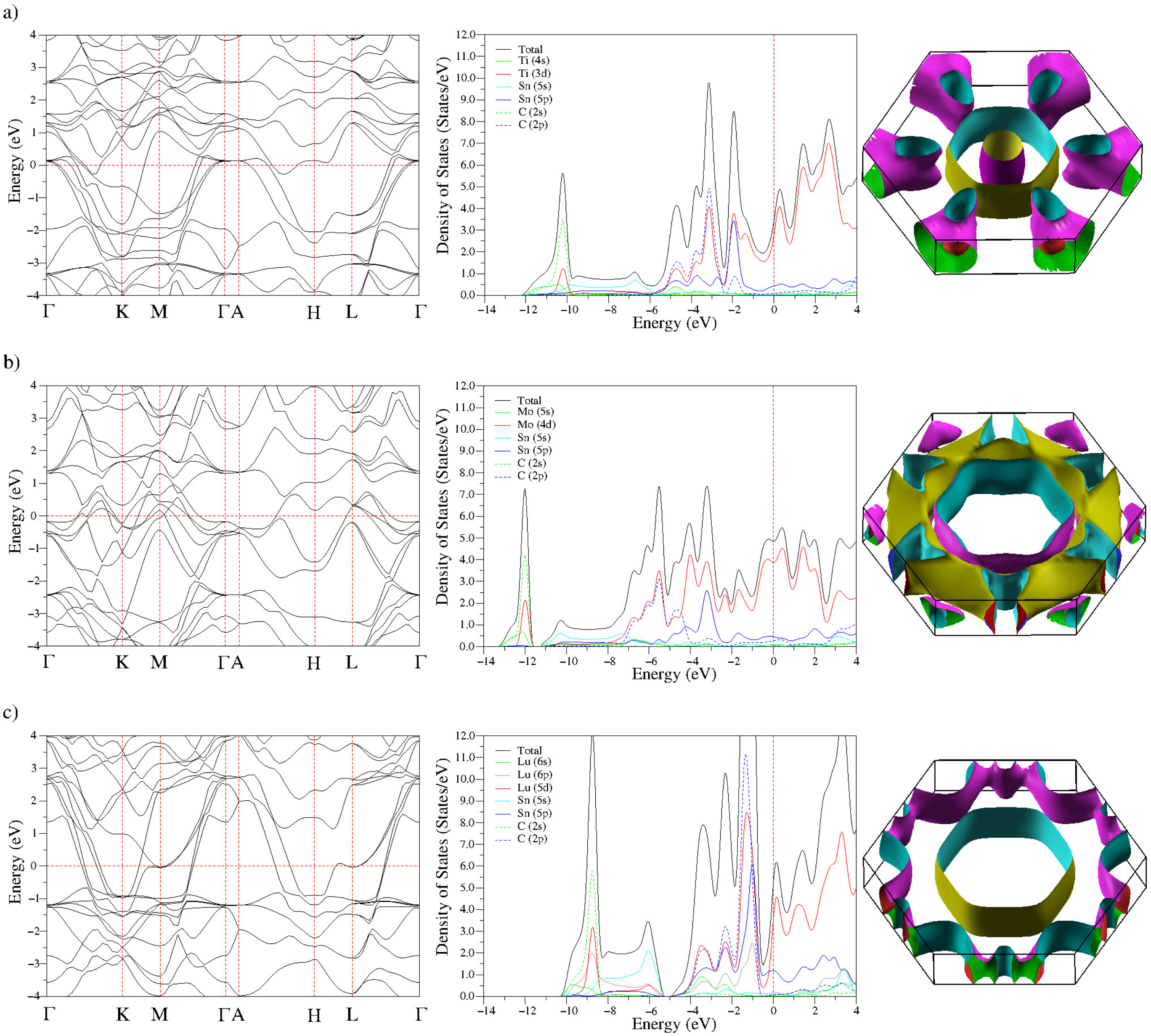

FIG. S4. The electronic band structure, the total and atomic projected electronic local density of states and Fermi surface for the Hexagonal phase of M_2SnC (M: Ti, Mo and Lu)

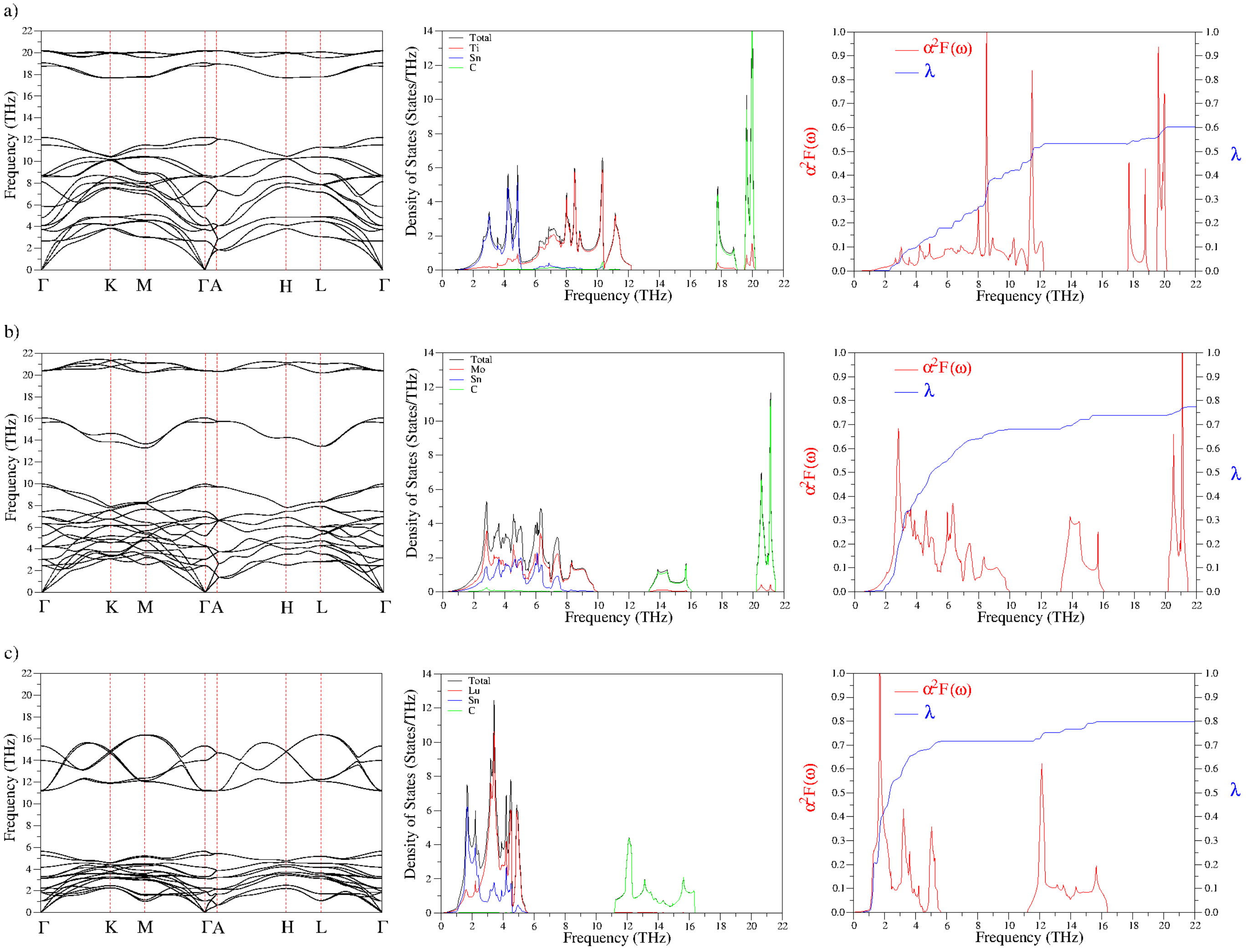

FIG. S5. Phonon dispersion curves, total, partial vibrational density of states and the calculated electron–phonon spectral function $\alpha^2F(\omega)$ (red line) and the variation of the electron–phonon coupling parameter (blue line) with rising frequency $\lambda(\omega)$ of M_2SnC (M: Ti, Mo and Lu)
